# Honokiol Crosses BBB and BCSFB, and Inhibits Brain Tumor Growth in Rat 9L Intracerebral Gliosarcoma Model and Human U251 Xenograft Glioma Model

**DOI:** 10.1371/journal.pone.0018490

**Published:** 2011-04-29

**Authors:** Xianhuo Wang, Xingmei Duan, Guangli Yang, Xiaoyan Zhang, Linyu Deng, Hao Zheng, Chongyang Deng, Jiaolin Wen, Ning Wang, Cheng Peng, Xia Zhao, Yuquan Wei, Lijuan Chen

**Affiliations:** 1 State Key Laboratory of Biotherapy and Cancer Center, West China Hospital, West China Medical School, Sichuan University, Chengdu, China; 2 State Key Laboratory Breeding Base of Systematic Research, Development and Utilization of Chinese Medicine, Chengdu University of Traditional Chinese Medicine, Chengdu, Sichuan, People's Republic of China; Queen Elizabeth Hospital, Hong Kong

## Abstract

**Background:**

Gliosarcoma is one of the most common malignant brain tumors, and anti-angiogenesis is a promising approach for the treatment of gliosarcoma. However, chemotherapy is obstructed by the physical obstacle formed by the blood-brain barrier (BBB) and blood-cerebrospinal fluid barrier (BCSFB). Honokiol has been known to possess potent activities in the central nervous system diseases, and anti-angiogenic and anti-tumor properties. Here, we hypothesized that honokiol could cross the BBB and BCSFB for the treatment of gliosarcoma.

**Methodologies:**

We first evaluated the abilities of honokiol to cross the BBB and BCSFB by measuring the penetration of honokiol into brain and blood-cerebrospinal fluid, and compared the honokiol amount taken up by brain with that by other tissues. Then we investigated the effect of honokiol on the growth inhibition of rat 9L gliosarcoma cells and human U251 glioma cells in vitro. Finally we established rat 9L intracerebral gliosarcoma model in Fisher 344 rats and human U251 xenograft glioma model in nude mice to investigate the anti-tumor activity.

**Principal Findings:**

We showed for the first time that honokiol could effectively cross BBB and BCSFB. The ratios of brain/plasma concentration were respectively 1.29, 2.54, 2.56 and 2.72 at 5, 30, 60 and 120 min. And about 10% of honokiol in plasma crossed BCSFB into cerebrospinal fluid (CSF). In vitro, honokiol produced dose-dependent inhibition of the growth of rat 9L gliosarcoma cells and human U251 glioma cells with IC_50_ of 15.61 µg/mL and 16.38 µg/mL, respectively. In vivo, treatment with 20 mg/kg body weight of honokiol (honokiol was given twice per week for 3 weeks by intravenous injection) resulted in significant reduction of tumor volume (112.70±10.16 mm^3^) compared with vehicle group (238.63±19.69 mm^3^, *P* = 0.000), with 52.77% inhibiting rate in rat 9L intracerebral gliosarcoma model, and (1450.83±348.36 mm^3^) compared with vehicle group (2914.17±780.52 mm^3^, *P* = 0.002), with 50.21% inhibiting rate in human U251 xenograft glioma model. Honokiol also significantly improved the survival over vehicle group in the two models (*P*<0.05).

**Conclusions/Significance:**

This study provided the first evidence that honokiol could effectively cross BBB and BCSFB and inhibit brain tumor growth in rat 9L intracerebral gliosarcoma model and human U251 xenograft glioma model. It suggested a significant strategy for offering a potential new therapy for the treatment of gliosarcoma.

## Introduction

Malignant gliomas account for approximately 70% of the 22,500 new cases of malignant primary brain tumor that are diagnosed in adults in the United States each year [1]. Gliosarcoma comprises about 2% of all glioblastomas and is a very aggressive tumor [2]–[5]. Despite optimal treatment, the median survival for patients with gliosarcoma is less than 12 months. Anti-angiogenesis is a promising approach for the treatment of gliosarcoma [6]–[9]. However, the blood-brain barrier (BBB) and blood-cerebrospinal fluid barrier (BCSFB) hampered the effects of both conventional and targeted therapies. Therefore, the necessary condition for drugs to act within brain is that drugs must cross the BBB and BCSFB. The BBB and BCSFB compose of capillary endothelial cells connected by tight junctions. Their main function is to be a physical and active barriers to restrict and regulate the penetration of compounds into and out from the brain to maintain brain homeostasis. The surface area of the BBB has been estimated to be 5000-fold greater than that of the BCSFB [10]. Therefore, the BBB is considered to be the major route for the uptake of endogenous and exogenous ligands into central nervous system.

Honokiol (3′,5-di-2-propenyl-1,1′-biphenyl-2,4′-diol), as a natural compound, has been isolated and identified from the stem bark of *Magnolia officinalis Rehd. et Wils.* (*Houpu* in Chinese). Honokiol has long been known to possess activities against oxidation [11], anxiety [12]–[15], depression [16], and in prevention and protection the brain from damage [17] in the central nervous system. Recent studies have shown that honokiol had also extensive anti-tumor efficacy in vitro and in vivo [18]–[21], especially, it exhibited strong anti-angiogenesis effects [22], [23]. In addition, honokiol was a potential strategy to overcome immunoresistance in glioma [24]. However, few studies, up to date, were reported on its abilities to cross BBB and BCSFB and the effects for the treatment of gliosarcoma in vivo. In this study, we hypothesized that honokiol might cross BBB and BCSFB, and exhibit its anti-tumor activity in rat 9L intracerebral gliosarcoma model and human U251 xenograft glioma model in vivo.

Two of several methods for assessing BBB penetration in vivo are widely used: determinations of brain/plasma ratio and measurement of the permeability×surface area product (PS) [25]. In our study, the methods determining the ratio of brain/plasma concentrations and the concentration of cerebrospinal fluid were used to evaluate whether honokiol could cross BBB and BCSFB. Then, intracerebral rat gliosarcoma model and xenograft human glioma model were established to evaluate the efficacy of honokiol via intravenous administration.

## Results

### Validation of HPLC Method and Honokiol Amount in Plasma

A HPLC method was developed to measure the amount in plasma, brain, cerebrospinal fluid and other tissues. The retention times of honokiol and internal standard were about 6.5 and 9.5 min by HPLC analysis, respectively. Linearity was determined using freshly prepared spiked plasma, CSF and tissues homogenate samples. The mean equations for the calibration curves of honokiol were y = 0.98x+0.63 (n = 5) with a correlation coefficients of 0.9989 in plasma, y = 0.53x+0.76 (n = 5) with a correlation coefficients of 0.9992 in CSF, y = 0.31x+0.19 (n = 5) with a correlation coefficients of 0.9991 in brain, y = 0.43x−0.68 (n = 5) with a correlation coefficients of 0.9996 in heart, y = 0.26x+0.35 (n = 5) with a correlation coefficients of 0.9986 in liver, y = 0.38x−0.56 (n = 5) with a correlation coefficients of 0.9985 in spleen, y = 0.41x+0.62 (n = 5) with a correlation coefficients of 0.9986 in lung and y = 0.36x+0.58 (n = 5) with a correlation coefficients of 0.9991 in kidney. The recoveries in plasma, CSF, brain and other tissues were between 86.23% and 104.62%. The intra-day RSD and inter-day were both less than 14.28%.

**Figure 1:**
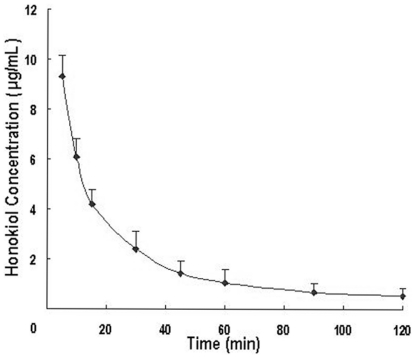
Mean plasma concentration-time curve of honokiol in rat plasma (The data from 0 min was omitted).

The mean plasma concentration-time curve of honokiol in rat plasma following intravenous administration of 20 mg/kg was shown in **Figure 1**. Each point with bar represented the mean+SD (n = 6). The pharmacokinetic parameters were presented in **Table 1**.

**Table 1:** The non-compartmental pharmacokinetic parameters of honokiol in rat plasma after intravenous administration (n = 6).

Parameters	T_1/2_(min)	C_max_(µg/mL)	AUC_0→t_(µg/mL*min)	AUC_0→∞_(µg/mL*min)	MRT_0→∞_(min)	V_d_(mL/g)	CL(mL/min/g)
Mean	41.37	9.30	262.28	285.99	40.74	4.18	0.07
SD	2.93	0.48	19.71	20.47	3.19	0.27	0.00

### BCSFB Penetration and CSF Distribution of Honokiol

**Table 2** displayed the amount of honokiol in CSF and the percentages of C_csf_/C_p_ (cerebrospinal fluid/plasma mean concentration percentages) at four time points. The results confirmed that honokiol could cross BCSFB into CSF and about 10% of its amount in plasma could do it. For example, 11.39% [(1.059 µg/mL/9.30 µg/mL)×100] of honokiol amount in plasma crossed BCSFB into CSF at 5 min post administration, 24.98% [(0.602 µg/mL/2.41 µg/mL)×100] of honokiol amount in plasma crossed BCSFB into CSF at 30 min, 9.05% [(0.095 µg/mL/1.05 µg/mL)×100] of honokiol amount in plasma crossed BCSFB into CSF at 60 min, and after 120 min (about third half-life time, T_1/2_ = 41.37 min), the percentage still reached 9.81 [(0.053 µg/mL/0.54 µg/mL)×100].

**Table 2:** Determined amount of honokiol in cerebrospinal fluid and brain at four time points after drug administration (n = 6 per time point).

	Concentration (mean ± SD)			
	5 min	30 min	60 min	120 min
Cerebrospinal fluid (µg/mL)	1.059±0.087	0.602±0.011	0.095±0.003	0.053±0.005
Brain (µg/g)	11.97±1.09	6.13±0.32	2.69±0.57	1.47±0.38
(C_csf_/C_p_)×100 (%)	11.39%	24.98%	9.05%	9.81%

### Assessment of BBB Penetration (Brain/Plasma Ratio) and Brain Distribution of Honokiol

Since the BBB is considered to be the major route for the uptake of endogenous and exogenous ligands into central nervous system, we investigated intensively the BBB penetration. **Table 2** showed the amount of honokiol in brain. The results revealed that honokiol could be easy to penetrate through the BBB into brain. Honokiol was detectable in rat brain and expeditiously achieved the peak value of 11.97±1.09 µg/g at 5 min post administration, and still kept a high concentration of 1.47±0.38 µg/g at 120 min (about third half-life time). The ratios of C_b_/C_p_ (brain/plasma concentration ratios), an important indicator to evaluate the brain uptake index [10], [25], were used to further evaluate BBB penetration of honokiol. The C_b_/C_p_ represented the ratio between the concentration in brain and the concentration in plasma at the same time point. Generally, the higher the ratio is, the greater the ability to cross the BBB is. As showed in **Table 3**, the ratios of C_b_/C_p_ at 5 min, 30 min, 60 min and 120 min were respectively 1.29, 2.54, 2.56 and 2.72, suggesting that honokiol was easy to enter the brain and had a good BBB penetration.

**Table 3:** Mean brain and plasma concentration of honokiol in rat after intravenous administration of 20 mg/kg (n = 6).

	5 min	30 min	60 min	120 min
Plasma (µg/mL)	9.30	2.41	1.05	0.54
Brain (µg/g)	11.97	6.13	2.69	1.47
C_b_/C_p_ (ratio)	1.29	2.54	2.56	2.72

### Comparison of Honokiol Distribution

The percentage of honokiol amount in all samples including plasma, brain and other tissues at 30 min after administration was calculated using the following equation:
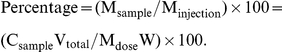
Where M_sample_ was the amount of honokiol (µg) in sample; M_injection_ was the injection amount of honokiol (µg); C_sample_ was the concentration of honokiol (µg/mL) in plasma or (µg/g) in tissues; V_total_ was the total volume of blood in animals which was 8% of body weight (mL) for plasma or was the total weight of every tissue (g) for tissues; M_dose_ was the injection dosage (µg/kg); W was the weight of every animal (kg).

**Figure 2:**
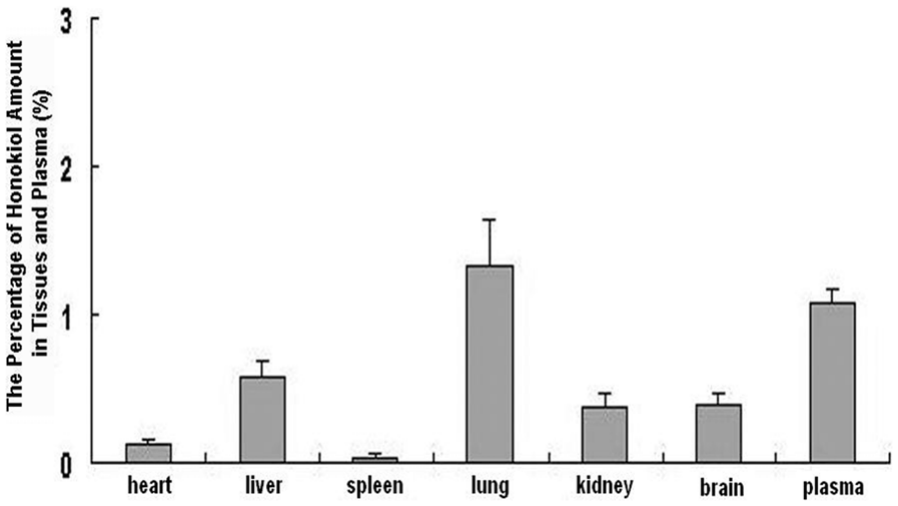
The percentage of honokiol amount in plasma, brain and other tissues at 30 min post-administration.

The results were shown in **Figure 2**. Data shown were presented as mean+SD (n = 6). The data revealed that the percentage of honokiol amount in brain was relatively high compared with other tissues and they were respective 0.39±0.08 in brain, 0.129±0.026 in heart, 0.58±0.11 in liver, 0.04±0.01 in spleen, 1.33±0.32 in lung, 0.37±0.09 in kidney and 1.09±0.08 in plasma. It was proved indirectly that honokiol could cross BBB into brain and kept a high amount in brain.

### Growth Inhibition of Honokiol against 9L Gliosarcoma and U251 Glioma Cells

**Figure 3:**
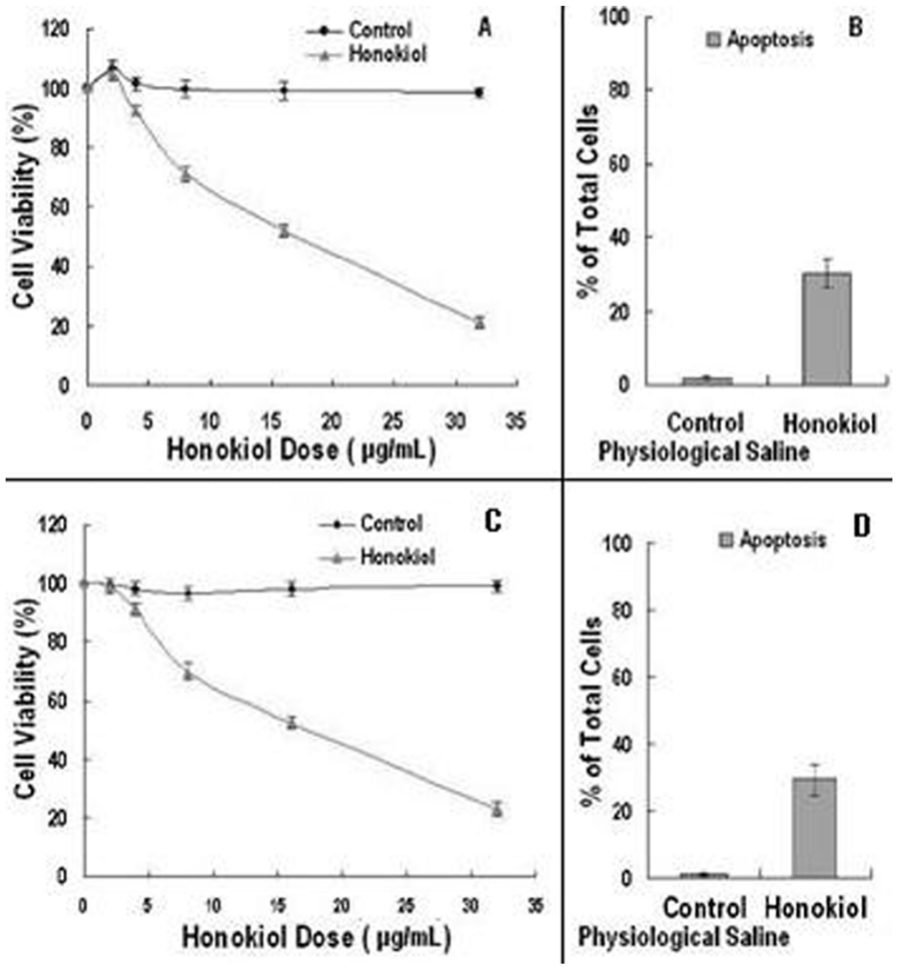
Honokiol inhibited the growth and induced apoptosis in rat 9L gliosarcoma and human U251 glioma cells. A and C, Dose-dependent inhibition of 9L gliosarcoma and U251 glioma cells growth, respectively: cells were exposed to various doses of honokiol for 24 h; B and D, Apoptosis of 9L gliosarcoma and U251 glioma cells, respectively: cells were treated with 16 µg/mL honokiol for 24 h.

The effects of presence or absence of honokiol on rat 9L gliosarcoma and human U251 glioma cells proliferation for 24 h were investigated. Honokiol reduced the rat 9L gliosarcoma and human U251 glioma cells number with IC_50_ of 15.61 µg/mL and 16.38 µg/mL, respectively. Honokiol produced dose-dependent inhibition of the growth of rat 9L gliosarcoma cells (**Figure 3, A**) and human U251 glioma cells (**Figure 3, C**) in culture. Data were expressed as mean ± SD (n = 3 at each dose).

### Effect of Honokiol on Apoptosis in 9L Gliosarcoma and U251 Glioma Cells

Apoptosis was measured using the sub-G_1_ DNA content determined via flow cytometry. Cells in the sub-G_1_ phase were considered to be apoptotic. The apoptosis rates were respectively 30.21±3.98% and 29.36±4.51% in rat 9L gliosarcoma cells (**Figure 3, B**) and human U251 glioma cells (**Figure 3, D**) when treated with 16 µg/mL honokiol for 24 h. Data were expressed as mean ± SD (n = 3).

### Effect of Honokiol on the Growth of 9L Gliosarcoma and U251 Glioma

**Figure 4:**
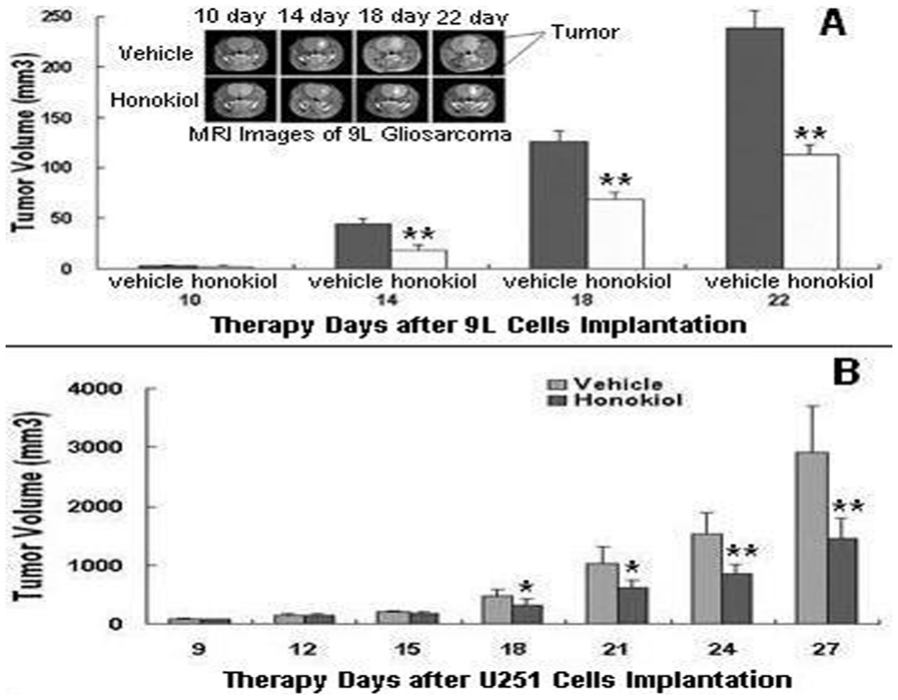
Honokiol decreased rat 9L intracerebral tumor and human U251 glioma growth in vivo. A for rat 9L gliosarcoma model; B for human U251 glioma xenograft model (*, *P*<0.05; **, *P*<0.01).

In rat 9L intracerebral gliosarcoma model, the tumor volumes and MRI images were measured every 4 days for 22 days by 3-T unit MRI. The tumor volume and some representative MRI images were indicated in **Figure 4, A**. Data shown were presented as mean ± SD of six animals per group (*, *P*<0.05; **, *P*<0.01). There was no statistical difference between vehicle group (2.52±1.46 mm^3^) and honokiol group (2.36±1.39 mm^3^) at 10-day treatment in tumor volumes. However, after 14-day of initiate treatment, honokiol had begun to present the capability of inhibiting the growth of 9L tumor (18.28±4.92 mm^3^) as compared with the vehicle group (43.92±5.07 mm^3^) (*P* = 0.000). After18-day of initiate therapy, tumor volumes in honokiol group rats were 68.95±6.52 mm^3^ *versus* 125.70±11.58 mm^3^ in vehicle group rats (*P* = 0.000), with 45.15% inhibiting rate of tumor volume. After 22-day of initiate treatment, honokiol group rats showed significantly lower tumor volumes (112.70±10.16 mm^3^) compared with vehicle group rats (238.63±19.69 mm^3^) (*P* = 0.000), with 52.77% inhibiting rate of tumor volume. At this time, all of the rats in vehicle group had large tumor volumes close to 250 mm^3^. The results demonstrated that honokiol was effective to inhibit tumor growth in rat 9L intracerebral gliosarcoma model.

In human U251 xenograft glioma model, the tumor volumes of nude mice were measured every 3 days for 27 days. The tumor volume were shown in **Figure 4, B**. Data shown were presented as mean ± SD of six animals per group (*, *P*<0.05; **, *P*<0.01). There were statistical differences between vehicle group and honokiol group after 18-day treatment in tumor volumes. The data of honokiol group *versus* vehicle group were respectively 318.33±105.05 mm^3^ *versus* 473.52±109.15 mm^3^ for 18-day of initiate treatment (*P* = 0.031), 615.02±124.10 mm^3^ *versus* 1034.81±279.58 mm^3^ for 21-day (*P* = 0.012), 850.32±107.78 mm^3^ *versus* 1527.20±365.42 mm^3^ for 24-day (*P* = 0.004) and 1450.83±348.36 mm^3^ *versus* 2914.17±780.52 mm^3^ for 27-day (*P* = 0.002) with 50.21% inhibiting rate of tumor volume.

### Effect of Honokiol on the Survival Time of 9L Gliosarcoma and U251 Glioma

**Figure 5:**
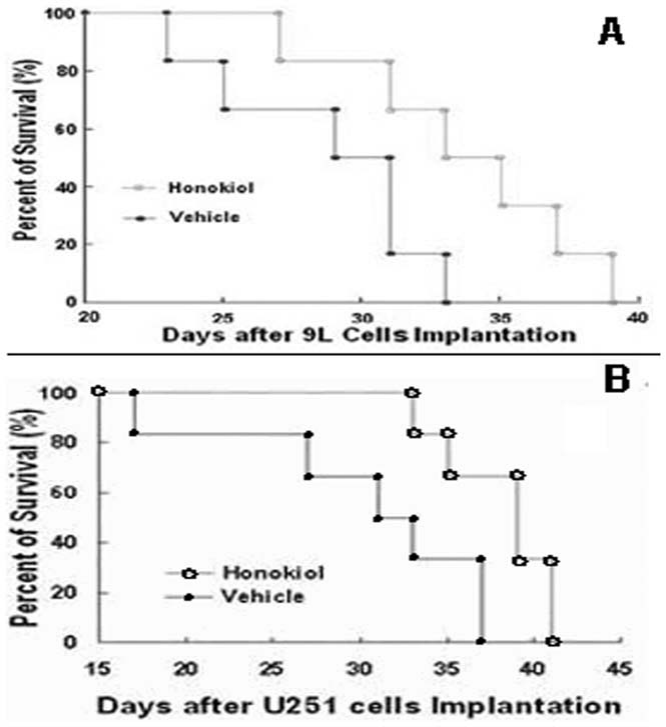
Honokiol prolonged the survival time of 9L intracerebral gliosarcoma rats and human U251 glioma xenograft nude mice. A for rat 9L gliosarcoma model; B for human U251 glioma xenograft model.

In rat 9L intracerebral gliosarcoma model, survival of rats in honokiol group was prolonged compared with that in vehicle group, and the mean survival time in honokiol group were 33.67±1.76 days post tumor implantation, however, 28.67±1.59 days in vehicle group. All rats in vehicle group died or their tumor volume increased to 250 mm^3^ at 33 days after 9L cells implantation. In contrast, honokiol treated group resulted in a significant increase in life span (*P*<0.05, by log rank test) and 50% rats survived at 33 days (**Figure 5, A**). Data shown were presented as mean ± SD of six animals per group. The 5-day growth delay observed for this very aggressive tumor was exciting and highly significant in light of the dismal prognosis of patients with brain tumors, being equivalent to a bout 4-month extension in life for a patient with a 1-year life expectancy [26], [27]. The results demonstrated that honokiol was effective to prolong survival time in rat 9L intracerebral gliosarcoma model. These properties of honokiol suggested additional investigation of its therapeutic potential in gliosarcoma in the clinic.

In human U251 xenograft glioma model, honokiol could prolong the survival of nude mice as the same above. Honokiol group resulted in a significant increase in life span (*P*<0.05, by log rank test) and 66.7% mice survived at 37 days, however, all mice in vehicle group died or their tumor volume increased to 4000 mm^3^ after U251 cells implantation at this time (**Figure 5, B**). Data shown were presented as mean ± SD of six animals per group.

### Inhibition of Angiogenesis

**Figure 6:**
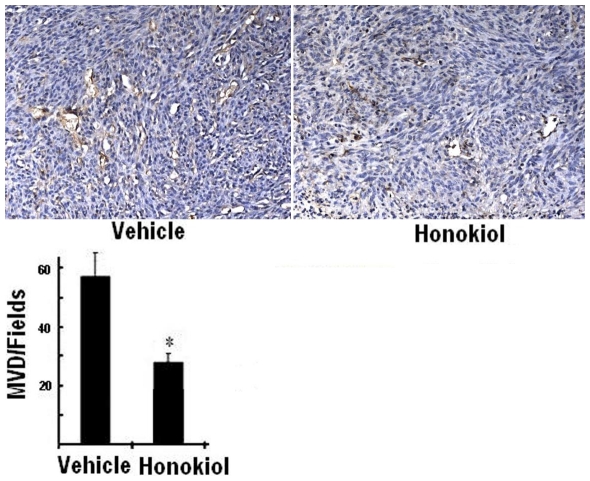
Tumor angiogenesis from human U251 glioma xenograft nude mice was assessed using immunohistochemical staining with anti-CD31 antibody. Microvessel counting was performed at 200×. Significantly reduced numbers of blood vessels in tumors treated with honokiol in comparison with vehicle. Data represented the mean ± SD of microvessels per high-power field (*, *P*<0.05 vs. vehicle).

Angiogenesis within tumor tissues from human U251 glioma xenograft nude mice was assessed in tumor sections stained with an antibody reactive to CD31 and quantified as described in Materials and Methods. The results were shown in **Figure 6**. Compared to vehicle group, honokiol group resulted in apparent inhibition of angiogenesis in tumors. The numbers of blood vessels treated with vehicle and honokiol were 58.6±9.1 and 29.1±2.3, respectively.

### Toxicity Evaluation

**Figure 7:**
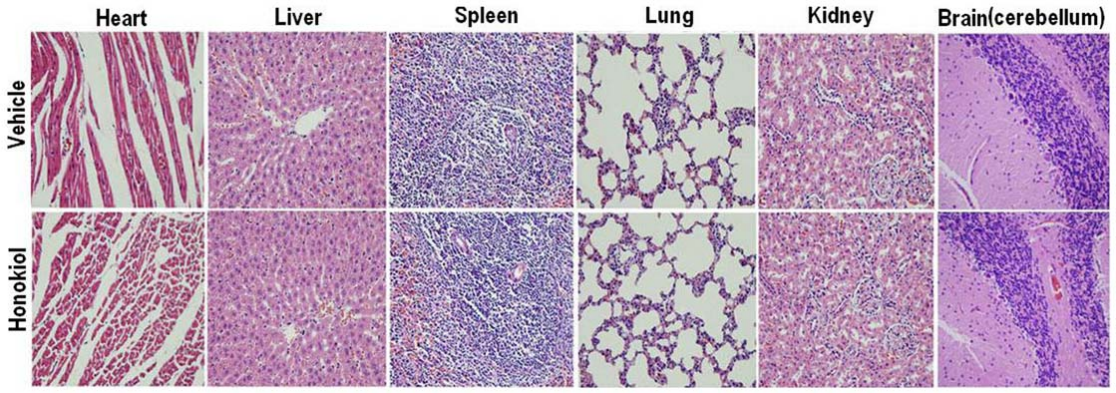
Histological staining of organs of rats with vehicle and high-dose honokiol. Rats were injected via caudal vein at a dose of 80 mg/kg body weight once a day for 14 days.

Toxicity of honokiol was evaluated in rats by intravenous administration with low-dose, middle-dose and high-dose once a day for 14 days. Body weight, mean daily food intake, hematological values, serum biochemical values and tissue pathologic changes of rats were investigated. The results showed that no significant differences were observed in body weight (**Table 4**), mean daily food intake(**Table 5**) hematological values(**Table 6**), serum biochemical values (**Table 7**) and tissue pathologic changes (**Figure 7**) between honokiol-individual dose and vehicle group. There were also no significant honokiol-dose-related differences.

**Table 4:** The effect of intravenous administration with vehicle or honokiol on the body weight of rats and data represented the mean ± SD (n = 5), the unit was g.

Time	Vehicle	Honokiol(20 mg/kg)	Honokiol(40 mg/kg)	Honokiol(80 mg/kg)
Day 0	169.1±5.3	167.8±4.9	174.1±4.2	168.7±3.6
Day 2	178.3±7.4	176.1±8.3	179.2±5.7	175.9±4.7
Day 6	205.5±9.4	199.5±10.3	210.4±9.8	216.5±10.2
Day 9	235.1±12.6	226.3±12.1	229.8±11.5	231.6±13.7
Day 13	271.9±13.2	261.3±14.7	280.4±13.6	269.2±15.1
Day 15	255.3±12.9	249.8±15.2	263.1±14.2	251.7±15.8

**Table 5:** The effect of intravenous administration with vehicle or honokiol on the mean daily food intake of rats and data represented the mean (n = 5), the unit was g.

Time	Vehicle	Honokiol(20 mg/kg)	Honokiol(40 mg/kg)	Honokiol(80 mg/kg)
Day 2	21.8	20.7	21.5	22.1
Day 9	25.3	26.6	24.9	24.6
Day 13	27.1	27.9	28.0	27.6

**Table 6:** The effect of intravenous administration with vehicle or honokiol on hematology of rats and data represented the mean ± SD (n = 5).

Parameter	Vehicle	Honokiol(20 mg/kg)	Honokiol(40 mg/kg)	Honokiol(80 mg/kg)
WBC (10^9^/L)	3.64±0.71	3.56±0.78	4.03±1.11	3.73±1.21
NEU (10^9^/L)	0.299±0.118	0.342±0.213	0.356±0.181	0.371±0.180
LYM (10^9^/L)	3.32±0.61	3.11±0.86	3.55±1.02	3.43±1.03
MONO (10^9^/L)	0.042±0.023	0.039±0.023	0.046±0.031	0.048±0.029
EOS (10^9^/L)	0.032±0.011	0.031±0.015	0.034±0.015	0.029±0.011
BASO (10^9^/L)	0.003±0.004	0.003±0.001	0.005±0.006	0.005±0.003
NEU% (%)	8.72±1.98	10.01±4.31	9.89±3.92	9.91±3.92
LYM% (%)	90.1±2.7	88.6±5.7	88.5±5.6	88.9±5.2
MONO% (%)	1.358±0.586	1.137±0.962	1.801±1.972	1.153±0.881
EOS% (%)	0.841±0.139	0.851±0.314	0.871±0.367	0.724±0.286
BASO% (%)	0.120±0.075	0.095±0.085	0.153±0.089	0.127±0.080
RBC (10^12^/L)	6.36±0.39	6.61±0.38	6.41±0.29	6.28±0.22
HGB (g/L)	138±9	131±8	135±6	128±8
HCT (%)	41.2±1.3	41.3±1.4	41.2±1.5	40.9±1.3
MCV (fL)	64.6±1.9	64.1±2.1	64.0±1.8	64.2±1.6
MCH (pg)	20.8±0.8	20.8±0.7	20.5±0.6	20.7±0.7
MCHC (g/L)	324±5	322±6	321±8	320±7
RET (%)	3.56±0.91	3.63±0.87	3.43±0.92	3.49±0.69
PLT (10^9^/L)	1134±123	1143±98	1161±126	1150±172
PT (sec)	8.2±0.4	8.1±0.5	8.2±0.2	8.2±0.2
APTT (sec)	17.2±1.5	17.3±1.9	17.2±1.5	17.1±1.6

**Table 7:** The effect of intravenous administration with vehicle or honokiol on serum biochemistry of rats and data represented the mean ± SD (n = 5).

Parameter	vehicle	Honokiol(20 mg/kg)	Honokiol(40 mg/kg)	Honokiol(80 mg/kg)
ALP (U/L)	171.3±65.1	159.2±70.9	149.8±59.8	160.1±63.9
ALT (U/L)	43.9±8.2	44.9±6.7	45.9±5.2	45.5±6.3
AST (U/L)	112.8±17.5	118.6±20.2	113.9±18.4	112.3±19.2
CK (U/L)	571±189	567±169	552±187	498±195
Urea (mmol/L)	5.01±0.91	4.71±0.67	4.55±1.01	4.73±0.96
Crea (µmol/L)	16.3±1.8	15.9±1.2	15.6±1.7	16.0±1.9
TP (g/L)	56.5±1.9	56.1±2.2	56.9±2.3	57.0±2.8
ALB (g/L)	40.2±1.6	40.1±1.7	41.2±1.6	41.3±2.0
A/G	2.60±0.65	2.51±0.41	2.60±0.41	2.49±0.71
GLU(mmol/L)	6.31±0.76	6.42±0.87	6.25±0.52	6.68±0.56
TBIL (µmol/L)	2.0±0.7	2.1±0.6	1.9±0.8	1.8±0.8
CHOL (mmol/L)	1.41±0.23	1.42±0.25	1.41±0.49	1.42±0.36
TG (mmol/L)	0.46±0.36	0.39±0.26	0.41±0.25	0.40±0.28
K^+^ (mmol/L)	3.99±0.43	3.91±0.18	3.87±0.28	4.03±0.85
Na^+^ (mmol/L)	139.2±1.5	138.9±2.1	139.8±1.8	140.2±1.9
Cl^−^ (mmol/L)	100.3±1.5	99.8±1.7	100.1±1.1	99.6±2.0

## Discussion

In this study, we had investigated whether honokiol could cross the BBB and BCSFB for the first time. The mean amount of honokiol in brain and CSF ranged from 11.97 to 1.47 µg/g and from 1.059 to 0.053 µg/mL, respectively. The amount of honokiol in brain and CSF changed up and down along with that in plasma. About 10% of honokiol amount in plasma crossed BCSFB into CSF. The BBB permeability of honokiol was measured by the ratio of C_b_/C_p_. We found that the ratios of C_b_/C_p_ reached immediately the max value at 30 min post administration and kept a steady-state level after 30 min. The level was 2∼3. In fact, most of anti-tumor drugs including paclitaxel were difficult to cross BBB and the ratio of C_b_/C_p_ was further less than 0.2 [28]. Our results demonstrated that honokiol had the preferable abilities of BBB and BCSFB penetrations, and the abilities may contribute to its suitable lipophilicity when honokiol was transported by passive diffusion, or its specific transport mechanisms when honokiol was transported by transporter. If honokiol was transported by transporter, the binding between membrane transport protein and honokiol would do not achieve saturation at the beginning of time. When the binding achieved saturation, the transport ability of membrane transport protein would keep balance. Therefore, the ratios of C_b_/C_p_ kept a steady-state level after 30 min.

The results of comparison of honokiol distribution in plasma, brain and other tissues showed that the order of percentage of honokiol amount was followings: lung>plasma>liver>brain>kidney>heart>spleen. The percentage ratios of lung to brain and liver to brain were respectively about 3.41–fold (1.33/0.39) and 1.49–fold (0.58/0.39). And the percentage value in brain was higher compared to other tissues. It was a surprise finding that the percentage value in brain was high and was close to that in liver which was the largest internal organ. Meanwhile, the results suggested that honokiol might possess a potential therapeutically strategy against liver cancer and lung cancer except brain cancer. In addition, in previous study the distribution of cisplatin, as one of malignant gliomas treatment drugs, showed that cisplatin accumulated mainly in kidney, liver, lung and heart [29]. From the above results, we could gain a conclusion that the two drugs, whether honokiol or cisplatin, mainly accumulated in lung, liver and kidney. The reasons might be summarized as follows: 1). Lung had a spongy texture and was honeycombed with epithelium having a much larger surface area in total than the outer surface area of the lung itself, which might make large quantities of drugs accumulate in lung. 2). Capillaries were the smallest, but most abundant blood vessels, where the exchange of gases between the blood and the tissues occured. The more capillaries around tissues were, the more amounts of drugs in tissues might be. Tissues such as liver and kidney had extensive capillary network, therefore, it might make large quantities of drugs accumulate in liver and kidney. The difference between honokiol and cisplatin in biodistribution was that the percentage value of honokiol amount in brain, which also had extensively specific capillary network named BBB and BCSFB, was high, however, the percentage value of cisplatin amount in brain was few. It might be concerned with abilities of crossing BBB and BCSFB of drugs.

Malignant glioma cells such as 9L and U251 are highly aggressive tumor cells of the central nervous system [27], [30]. Previous studies had shown that mean survival of patients with malignant gliomas (glioblastoma multiforme or gliosarcoma) after surgery and radiotherapy was approximately 10 months, and chemotherapy had been relatively ineffective in extending survival due to their poor penetration through the BBB and BCSFB [31]. Cisplatin, as one of cytotoxic drugs, had only weak effect in rat 9L gliosarcoma model. However, cisplatin in combination with antiangiogenic agent thalidomide improved the treatment efficacy for the intracranial tumors, and increased the survival time from 18 days for the untreated controls to 20.5 days [29]. Dexamethasone, as a glucocorticoid hormone and antiangiogenic agent, could decrease tumor volume compared to control group with about 60% maximum inhibiting rate in rat 9L intracerebral gliosarcoma model [32]. In our study, an orthotopic rat 9L intracerebral gliosarcoma model and human U251 xenograft glioma model were established to evaluate the efficacy of honokiol via intravenous administration. The results revealed that honokiol was an antiangiogenic agent and could well contribute anti-tumor activity by inhibiting tumor growth and prolonging survival time. Honokiol significantly reduced tumor volume with high inhibiting rate and significantly improved the survival time compared to vehicle group.

Due to the existence of specific efflux transporters, brain tumors are easy to be resistant to chemotherapy. P-glycoprotein played an important role in restricting access of substrate drugs across the BBB in to brain [33], [34]. Previous studies have proved that some anti-tumor drugs including vincristine and cyclosporine A have the low BBB and BCSFB penetration into brain ascribe to the existence of P-glycoprotein [33], [35]. Therefore, it is urgent to develop a chemotherapy agent of brain tumor without resulting in resistance. The high C_b_/C_p_ ratios and good orthotopic anti-gliosarcoma activity of honokiol suggested that honokiol might have the abilities to escape the effect of P-glycoprotein and cross BBB into brain, and contribute to the anti-tumor activity in brain tumors. Therefore honokiol might be a potential therapeutically strategy against gliosarcoma.

## Materials and Methods

### Ethics and Statement

This study was carried out in strict accordance with the recommendations in the Guide for the Care and Use of Laboratory Animals of the National Institutes of Health. The protocol was approved by our Institutional Animal Care and Use Committee of the Sichuan University in China (Permit Number: 20100318). All surgery was performed under chloral hydrate anesthesia, and all efforts were made to minimize suffering.

### Reagents, Animals, Cell Line and Cell Culture

Honokiol (purity ≥98%) was separated and purified in our laboratory, as reported previously [36]. The lipopolysaccharide (LPS) contamination of honokiol was directly confirmed by Limulus Amebocyte Lysate (LAL) test and the value was less than 0.25 EU per mg of honokiol. Diphenyl (purity ≥99%, internal standard) was purchased from the National Institute for the Control of Pharmaceuticals and Biological Products (Chengdu, China). All chemicals and solvents were American Chemical Society (ACS) analytical grade or HPLC grade. Male Sprague-Dawley rats (8∼10 weeks old, 220∼260 g, n = 30) and male Sprague-Dawley rats (5∼6 weeks old, 160∼180 g, n = 20) were obtained from the Laboratory Animal Center of Sichuan University in China. Male Fisher 344 rats (200∼220 g, n = 12) and female athymic nude mice (6∼8 weeks old, 18∼22 g, n = 24) were purchased from the Animal Institute of the Chinese Medical Academy (Beijing, China).

The rat 9L gliosarcoma and human U251 glioma cell lines were purchased from American Type Culture Collection (Rockville, MD, USA) and passaged in our laboratory for about 2 months after receipt. Cells were cultured in DMEM supplemented with 10% heat-inactivated fetal calf serum, penicillin (10 IU/mL), streptomycin (10 µg/mL) and 10% nonessential amino acids, and maintained at 37°C in a humidified incubator containing 5% CO_2_ and harvested by trypsinization at 70% to 80% confluence in log-phase growth on the day of tumor injection (day 0).

### Measurement of Honokiol Amount After Intravenous Administration in Plasma

Male Sprague-Dawley rats ((8∼10 weeks old, 220∼260 g, n = 6) were anesthetized with chloral hydrate and then a polyethylene tube (0.28 mm, I.D., 0.61 mm, O.D.) was inserted into the right femoral artery of rat. The rats were placed in cages and allowed to recover from anesthesia for more than 1 h. The honokiol dissolved in mixture of polyethoxylated castor oil and ethanol (1∶1, v/v) was administered to the rats at a single dose of 20 mg/kg body weight (concentration of honokiol: 4 mg/mL; injected volume of solution per rat: 1.1∼1.3 mL) via caudal vein. Then about 0.20 mL of blood samples were collected through the cannulated tube at designated time for 0, 5 min, 10 min, 15 min, 30 min, 45 min, 1 h, 1.5 h and 2 h into tubes containing heparin sodium. After 2 h, the rats were euthanized via CO_2_ asphyxiation. Plasma samples were obtained after immediate centrifugation of blood at 3000 rpm for 10 min at 4°C. Plasma samples were prepared as described previously [37]. Diphenyl was selected as internal standard and 10 µL of prepared samples was directly injected into HPLC system for analysis.

### Perfusion Method

The honokiol was also administered to other male Sprague-Dawley rats ((8∼10 weeks old, 220∼260 g, n = 24) at a single dose of 20 mg/kg body weight via caudal vein. The time for respective 5 min, 30 min, 60 min and 120 min (Six rats per time point) post administration was designated to collect the cerebrospinal fluid (CSF), brain and other tissues after cardiac perfusion through left ventricle using the perfusion method described previously [38] to determinate exactly the amount of honokiol. Rats were perfused with Krebs-Henseleit buffer (118 mM NaCl, 14.7 mM KCl, 2.5 mM CaCl_2_, 1.2 mM MgSO_4_, 1.2 mM KH_2_PO_4_, 25 mM NaHCO_3_, 10 mM D-glucose, pH 7.4) containing heparin (5 U/mL). This buffer was bubbled with a mixture of 95% O_2_ and 5% CO_2_, filtered through Millex HV filter (Millipore, Bedford, MA), incubated for about 30 min on ice. Peristaltic pump speed was set at 30 mL/min and time taken for perfusion was 5 min.

### Evaluation of BBB and BCSFB Penetration of Honokiol in Vivo

**Figure 8:**
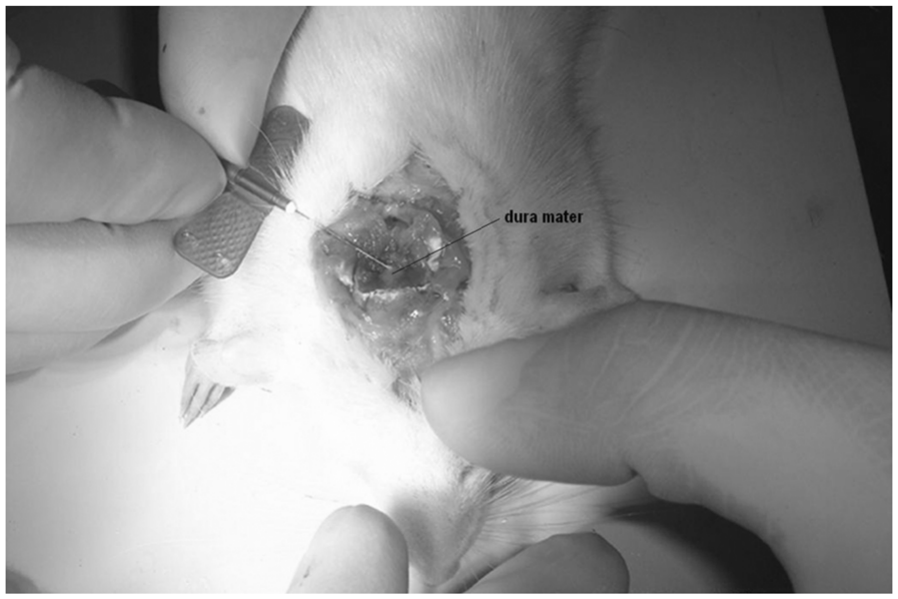
Collection of CSF from cisterna magna with a rat.

Following perfusion, the rats were immediately mounted in a ligneous device. The back of the neck and base of the skull were shaved and disinfected with 70% ethyl alcohol. A midline incision was made beginning at between the ears and ending approximately 2 cm caudally. The fascia was retracted and superficial muscles were dissected. A retractor was placed with the spring side pointing in rostral direction. Separation of the superficial muscles exposed an underlying layer of muscles which could be easily separated along the midline by blunt dissection, exposing the atlanto-occipital membrane. In order to gain better access to the atlanto-occipital membrane, the incisor bar was lowered on the animal's head, inducing a downward curvature of about 45°C from horizontal. A small slit was made along the midline of the membrane and the incision was then enlarged to expose the underlying dura mater. Immediately a needle attached to 1 mL syringe was carefully inserted at a 30° angle to the dura, from the caudal end of the incision. The needle was inserted with the bevel of the needle faced up initially. When the bevel was covered by the dura, the needle was then gently shifted so that the angle of insertion was parallel with the dura and the bevel was turned away from the dura. Once parallel with the dura, the needle was inserted approximately 1 mm farther beneath the dura to ensure that it did not come out during CSF collection. Positioning of the needle was chosen to prevent damage of vasculature on the pia. Finally, aspiration of CSF was achieved by pulling back the syringe plunger, which was completed in less than 1 min. The operation was shown in **Figure 8**. Approximately 0.1 mL of CSF was obtained and stored at −80°C until analysis by HPLC. The rats were then decapitated and forebrains of the rats were separated from the cerebellum and medulla oblongata. After removal of the pia mater and choroid plexus, the brains of rats were collected, lightly blotted to remove any excess fluid, weighted and stored at −80°C until analysis by HPLC.

Brain samples were weighed accurately and homogenized in PBS (pH 7.4, 250 mg/mL). The analytical process of CSF and brain homogenized samples kept the same as that of plasma samples. Dissimilarly, the volume (1 mL) of brain homogenized samples was 10-fold compared to plasma samples (0.1 mL).

### Honokiol Amount Taken Up by Other Tissues

Similar to the described above, after brains were collected, heart, liver, spleen, lung and kidney were also collected at 30 min time point. The analytical process of these tissues samples kept the similar to that of brain samples (Some lung samples needed to be diluted).

### HPLC Analysis

Chromatographic analyses were performed with an Alliance 2965 HPLC system (Waters, Milford, MA, USA) consisting of a column heater and an autosampler, and detection was carried out by using UV detector at 209 nm. Chromatographic separation of honokiol was carried out on a reversed-phase column (Atlantis C18 column, 150×4.6 mm, 5 µm, Waters). The column temperature was maintained at 28°C. Samples were eluted using acetonitrile and water (60∶40, V/V) at a flow rate of 1.0 mL/min.

### Growth Inhibition Assays

In vitro growth inhibition of honokiol for rat 9L gliosarcoma and human U251 glioma cells was assessed using an MTT assay. Briefly, cells were seeded in a 96-well plate at a plating density of 5×10^4^/mL and cultured for 24 h to allow them to adhere to the plate. Harvested cells were exposed in presence or absence with different concentrations of honokiol (0∼32 µg/mL) in fresh DMEM medium. The plates were placed at 37°C for 24 h. Then cells were added to 20 µL of MTT (5 mg/mL) and cultured for 3 h at 37°C. The supernatant was removed, the plate was reloaded with 150 µL of DMSO, and the absorbance was measured at 570 nm using a Spectramax M5 Microtiter Plate Luminometer (Molecular Devices). The absorbance value of untreated cells was considered to be 100%. IC_50_ was defined by the concentration that caused a 50% absorbance decrease in treated cells compared with untreated cells. Three replicates of each well for each treatment dose were performed.

### Assessment of Apoptosis by Flow Cytometry

To quantitative assessment of apoptosis, flow cytometric analysis was applied to identify sub-G_1_ cells/apoptotic cells and measure the percentage of sub-G_1_ cells. Rat 9L gliosarcoma and human U251 glioma cells were seeded in a 6-well plate and treated with physiological saline and honokiol (16 µg/mL, about IC_50_) for 24 h, respectively. Then cells were collected, washed with PBS, and suspended in 1 mL hypotonic fluorochrome solution containing 50 µg of propidium iodide/mL in 0.1% sodium citrate plus 0.1% Triton X-100. The cells were analyzed using a flow cytometer (ESP Elite, Beckman-Coulter, Miami, FL). The numbers of apoptotic cells appearing in the cell cycle distribution were estimated using Listmode software. The experiments were repeated thrice.

### Intracerebral Tumor Model and Xenograft Tumor Model

The orthotopic intracerebral gliosarcoma model was established to evaluate directly the efficacy and assess indirectly the blood-brain barrier penetration of honokiol as described previously [26], [39]–[42]. All Fisher 344 rats (n = 12) were anesthetized and then the orthotopic gliosarcoma allograft was generated by intracerebral injection of rat 9L cells (5×10^4^ cells in 10 µl serum-free DMEM). The heads of the rats were immobilized in a stereotactic frame, shaved and disinfected with 10% povidone iodine. After a midsagittal scalp incision to expose the skull, a small burr hole was drilled at 3 mm to the right of the sagittal suture and 1 mm anterior to the coronal suture. A 10-gauge needle connected to a microsyringe was attached to a stereotactic manipulator. The needle was inserted into a depth of 5 mm under cerebral dura mater, and then the cell suspension was injected into the caudatum slowly over a 5-min period. The needle was left in this place for 5 min to allow for diffusion before slow withdrawal. The burr hole in the skull was sealed with sterile bone wax and the incision was closed.

The human U251 xenograft glioma model was also established to further prove the efficacy of honokiol as described previously [43], [44]. The human U251 glioma cells (5×10^5^ cells) were injected s.c. into the right flanks of athymic nude mice (n = 24).

### Assessment of Anti-Tumor Activity in Vivo

The orthotopic intracerebral tumor rats were randomly and equally divided into two groups (6 rats for each group) when 9L cells were stereotactic injected for seven days. The first group (vehicle group) was injected via caudal vein with vehicle solution consisting of 2.5% mixture of Cremophor EL and ethanol in 5% dextrose. The second group (honokiol group) was injected via caudal vein with honokiol (dissolved in vehicle solution) at a dose of 20 mg/kg body weight (concentration of honokiol: 4 mg/mL; injected volume of solution per rat: 1.0∼1.1 mL). Each group was given treatment twice per week for 3 weeks. Tumor volume was measured and calculated by 3-T unit magnetic resonance imaging (MRI) in West China Hospital (Chengdu, China) according to the area of tumor in each MR image slice every 4 days for 22 days. We examined the animals daily for survival until 40 days and scored them as dead when tumor volume increased to 250 mm^3^. The rats without death were euthanized via CO_2_ asphyxiation.

The xenograft U251 glioma nude mice were also randomly and equally divided into vehicle group and honokiol group (12 mice for each group) when tumors were palpable (about 30 mm^3^). Each group was given treatment via caudal vein administration twice per week for 3 weeks similar to the dosage and method above. Tumor volume was measured every three days with a caliper and calculated using the following formula: 0.523×length×width^2^. Six mice in each group were decapitated on the 27th day. Tumor, heart, liver, spleen, lung, kidney and brain were excised and fixed in 4% paraformaldehyde. Other six mice in each group were examined daily for survival until 45 days and scored as dead when tumor volume reached 4000 mm^3^. The mice without death were euthanized via CO_2_ asphyxiation.

### Immunohistochemistry for Microvessel

Tumor tissues from human U251 glioma xenograft nude mice at the end of treatments were paraffin embedded, cut into 4-µm sections, and stained using rabbit monoclonal antibody against mouse marker CD31 (BD Biosciences Pharmingen, San Diego, CA) to investigate the anti-angiogenesis activity of honokiol. The vessel density was determined by counting the number of microvessels per one high-power field [23].

### Evaluation of Toxicity

The toxicity evaluation of honokiol was performed as previously described with some modifications [45]. Male Sprague-Dawley rats (5∼6 weeks old, 160∼180 g, n = 20) were divided into four groups (5 rats in each group): vehicle group, low-dose group (20 mg/kg body weight), middle-dose group (40 mg/kg body weight) and high-dose group (80 mg/kg body weight). The rats were housed in individual wire cages in each group and administrated via caudal vein with vehicle or honokiol once a day for 14 days. During the experiment, body weight of every rat was measured at the 0 day, 2th day, 6th day, 9th day and 13th day. After the 14th administration, the rats were banned diet for 12 h and body weight of every rat was again measured at the15th day. The total food intake in each cage (each group) was measured at the 2th day, 9th day and 13th day during 24 h via weight loss method, respectively. And the mean daily food intake fo rat in each cage (5 rats in each cage) was calculated by averaging method. At the 15th day, all rats were anesthetized and sacrificed. Blood was collected into a tube and centrifugated (3000 rpm, 10 min), and then plasma samples were analyzed for homatological test including WBC (white blood cell count), NEU (number of neutrophils), NEU% (percent of neutrophils), LYM (number of lymphocytes), LYM% (percent of lymphocytes), MONO (number of monocytes), MONO% (percent of monocytes), EOS (number of eosinophils), EOS% (percent of eosinophils), BASO (number of basophils), BASO% (percent of basophils), RBC (red blood cell count), HGB (hemoglobin), HCT (hematocrit), MCV (mean corpuscular volume), MCH (mean corpuscular hemoglobin), MCHC (mean corpuscular hemoglobin concentration), RET (reticulocyte count), PLT (platelet count) using a blood cell counter (Hemavet 0950, CDC Technology, Irvine, CA), PT (prothrombin time), APTT (activated partial thromboplastin time) using a Coagulometer ACL 100 (Instrumentation Laboratory Co, Lexington, KY, USA). Serum samples were also obtained and examined for biochemical test including ALB (albumin), ALP (alkaline phosphatase), AST (aspartate aminotransferase), ALT (alanine aminotransferase), CHOL (cholesterol), Urea (blood urea-N), Crea (creatinine), GLU (glucose), TP (total protein), TBIL (total bilirubin), CK (creatine phosphokinase), Na (sodium), K(potassium), Cl (chloride), TG (triglyceride), and A/G (ratio of albumin to globulin) using a biochemical blood analyzer (Hitachi 7180, Hitachi, Japan). Tissues of heart, liver, spleen, lung, kidney and brain (cerebellum) were also collected and embedded in paraffin, cut into 4-µm sections for H&E staining.

### Statistical Analysis

The concentration-time data of honokiol was fitted by the Drug and Statistics (DAS) software, version 2.1.1, edited and published by the Mathematical Pharmacology Professional Committee of China. All statistical data were analyzed using the SPSS 13.0 software. Statistical comparisons were made with one-factor analysis of variance (ANOVA). For the survival time of animals, Kaplan-Meier curves were established for each group, and the survivals were compared by means of the log rank test. Student's *t*-test was used to calculate probability (*P*) values. In all test a *P* value of <0.05 was regarded as statistically significant. Experiments were performed at least in triplicate.
